# Impact of the Addition of Disaccharides on the Encapsulation of Chokeberry Polyphenols on Rice and Pea Proteins

**DOI:** 10.3390/molecules31020377

**Published:** 2026-01-21

**Authors:** Mirela Kopjar, Ivana Buljeta, Dubravko Pichler, Josipa Krezić, Nela Nedić Tiban, Anita Pichler

**Affiliations:** 1 Faculty of Food Technology Osijek, Josip Juraj Strossmayer University of Osijek, F. Kuhača 18, 31000 Osijek, Croatia; ivana.buljeta@hotmail.com (I.B.); nela.nedic@ptfos.hr (N.N.T.); anita.pichler@ptfos.hr (A.P.); 2 Water Supply-Osijek, Poljski put 1, 31000 Osijek, Croatia; dubravko.pichler@vodovod.com; 3 Faculty of Agriculture and Food Technology, University of Mostar, Biskupa Čule bb, 88000 Mostar, Bosnia and Herzegovina; josipa.krezic@aptf.sum.ba

**Keywords:** plant-based protein aggregates, sucrose, trehalose, chokeberry polyphenols, antioxidant potential

## Abstract

Promising approach for the expansion of the functional food sector is combining various ingredients with potential health benefits. The aim of this study was to create protein aggregates by freeze-drying encapsulation. Rice or pea proteins were used as carriers for encapsulation of chokeberry juice polyphenols. Additionally, disaccharides (sucrose and trehalose) were added to explore possible enhancement of encapsulation of polyphenols. Two methods were employed for complexation of ingredients prior to freeze-drying: one based on complexation of all ingredients at the same time and the other on complexation first of proteins with disaccharides and then with chokeberry juice. All parameters affected the binding of polyphenols on proteins. Total polyphenols, proanthocyanidins, individual polyphenols, and antioxidant potentials of created protein aggregates were determined. When rice protein was the main carrier, the addition of disaccharides caused a decrease in total polyphenols and proanthocyanindins contents (22.41–24.01 mg GAE/g and 6.36–7.28 mg PB2E/g, respectively) in comparison to aggregates without their addition (28.03 mg GAE/g and 8.57 mg PB2E/g, respectively). In the case of pea proteins, a different trend was observed. Aggregates without disaccharide addition had a lower amount of total polyphenols and proanthocyanindins (21.25 mg GAE/g and 5.56 mg PB2E/g, respectively) than those with disaccharide addition (21.42–26.44 mg GAE/g and 6.37–9.45 mg PB2E/g, respectively). Interactions between compounds were proven through IR spectra, and they included changes in amid structures, as well as hydrogen bonds and hydrophobic interactions. Such formulated plant-based protein aggregates can be used in the food industry for the enrichment of foods with polyphenols, incensement of antioxidant potential, and prolonging stability of products.

## 1. Introduction

Recently, the food industry has been targeting the expansion of the functional food sector through the development of functional additives by combining different ingredients with potential health benefits, as well as with different technological properties. Generally, on the one hand, polyphenols are known for their many potential health benefits (antioxidant, anti-inflammation, and anticancer properties; possible stabilization of the lipid profile, diabetes, and hyperglycemia; and protection from cardiovascular disorders), while on the other hand, they are known for their high instability under various conditions [1,2,3,4,5,6]. In this study, polyphenols of chokeberry juice were combined with plant proteins and disaccharides in order to create new functional food additives. In recent years, an appreciable nutritional chemical profile of chokeberries has been pointed out through several published reviews, underlining polyphenols as one of the major contributors to their potential health benefits [7,8,9,10]. Commonly, different products like juice, jams, spreads, sauces, wines, fruit teas, and dietary supplements are produced from chokeberries [11,12], but chokeberries also present a solid base for the production of functional food additives [7,8]. These fruits are especially rich in anthocyanins (such as cyanidin-3-galactoside and cyanidin-3-arabinoside), flavonols (quercetin-3-rutinoside), and phenolic acids (chlorogenic and neochlorogenic) [10,11,13].

Over the years, different encapsulation techniques obtained substantial focus in the food and pharmaceutical industries since one of their targets is the preservation of bioactive compounds, which are often rather unstable under divergent environmental conditions. For efficient encapsulation, the selection of an appropriate carrier is of high importance [14]. Implementation of the “delivery by design concept” can be used to resolve this rising question of stability of phenolic compounds. Substantial efforts are devoted to creating new formulations through combinations of polyphenols and different polymers. Combining polyphenols with protein matrices is one of the strategies in this direction, in particular plant-based solutions, i.e., in our study complexation of polyphenols with plant proteins and disaccharides. Over the years, the utilization of plant-based proteins as carriers for the encapsulation of active ingredients has been on the rise due to the existence of the hazard of animal-borne diseases. Relevant plant sources that are currently utilized for the extraction of proteins and are successfully applied in the encapsulation process are legume seeds, soybeans, corn kernels, peas, rice, wheat, quinoa, sunflowers, and pumpkin seeds. Isolated proteins, next to encapsulation efficiency, are used because of their affordable price, high accessibility, nutritional value i.e., profile of amino acids, and some techno-functional properties like emulsification and gelation [15,16,17,18,19,20,21,22,23,24,25]. Next to using a biopolymer as the main carrier, it is possible to apply additional ingredients that can increase the encapsulation of bioactive compounds; however, this mainly depends on interactions that can occur in such systems. For example, in a previous study, it was determined that the addition of disaccharide had an impact on the ionic gelation encapsulation of chokeberry juice polyphenols when they were added in the encapsulation mixture next to the alginate and alginate in combination with pullulan [26]. Thus, we aim to investigate the addition of a disaccharide on the encapsulation of polyphenols on a protein matrix by freeze-drying. Sucrose and trehalose were included in the complexation mixture alongside plant proteins. Both disaccharides are applied in the food industry. Sucrose is commonly utilized in the food industry as well as in households. Trehalose utilization rises through the years primarily due to bioprotective properties and non-toxicity [27], but also due to some other characteristics that are not ascribed to sucrose. Comparing the sweetness of those two disaccharides, trehalose has lower sweetness than sucrose, approximately 45% of sucrose. Substantially reduced cariogenic ability in comparison to sucrose is another feature of trehalose that is highly beneficial for children, accompanied by the exclusion of the laxative effect that other low-cariogenic sweeteners possess [28]. Taking into account that trehalose is slowly digested, which results in the decreased release of insulin in the human body in contrast to sucrose, a low glycemic index is also a relevant feature of trehalose [29,30,31,32].

The aim of this study was to create protein aggregates based on chokeberry juice polyphenols and plant-based proteins (rice or pea proteins) without and with the addition of disaccharides (sucrose or trehalose) during complexation. Additionally, two methods of complexation of the ingredients were investigated. In the first method, all ingredients were complexed at the same time, while in the second one, the first disaccharide was mixed with protein and then with chokeberry juice. Created protein aggregates were evaluated for their total polyphenols, proantocyanidins, and individual polyphenols. In addition, the antioxidant potential of the aggregates was estimated. In order to prove the binding of polyphenols on protein, as well as the binding of disaccharides and polyphenols on protein, IR spectra were recorded.

## 2. Results

### 2.1. Adsorption Capacity of Polyphenols on Protein Aggregates

For easier understanding of the results, Table 1 represents the samples coding.

**Figure 1:**
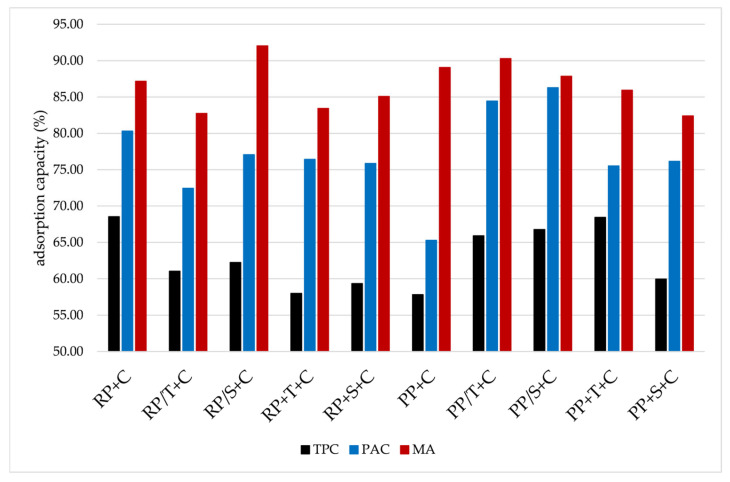
Adsorption capacity of total polyphenols (TPC), proanthocyanidins (PAC), and monomeric anthocyanins (MA) on protein aggregates (RP+C: complexation of rice proteins and chokeberry juice; RP/T+C: first complexation of rice proteins with trehalose and then with chokeberry juice; RP/S+C: first complexation of rice proteins with sucrose and then with chokeberry juice; RP+T+C: complexation of all ingredients (rice proteins, trehalose, and chokeberry juice) at the same time; RP+S+C: complexation of all ingredients (rice proteins, sucrose, and chokeberry juice) at the same time; PP+C: complexation of pea proteins and chokeberry juice; PP/T+C: first complexation of pea proteins with trehalose and then with chokeberry juice; PP/S+C: first complexation of pea proteins with sucrose and then with chokeberry juice; PP+T+C: complexation of all ingredients (pea proteins, trehalose, and chokeberry juice) at the same time; PP+S+C: complexation of all ingredients (pea proteins, sucrose, and chokeberry juice) at the same time.

The adsorption capacity of chokeberry polyphenols on rice and pea proteins, as well as the impact of disaccharide addition on the adsorption capacity, is presented in Figure 1. Selected proteins differ in their affinity for polyphenols, and results showed that disaccharide addition and their type had an impact on the adsorption of polyphenols. Higher adsorption capacity of total polyphenols was calculated for rice proteins (68.5%) in contrast to 57.8% for pea proteins. Proanthocyanidins followed a similar trend, i.e., higher adsorption capacity was determined on rice proteins (80.3%) than on pea (65.3%). Adsorption capacities of monomeric anthocyanins were similar for both proteins, 87% for rice and 89% for pea proteins. These results were used as control, and protein aggregates with disaccharides addition were compared to them. When all ingredients (proteins, disaccharides, and chokeberry juice) were complexed together at the same time, disaccharide addition enhanced the adsorption of total phenolics onto pea proteins, while the reverse effect was observed for rice proteins. Trehalose addition increased the adsorption capacity of total polyphenols on pea protein aggregates up to 68.4%, while sucrose addition increased it up to approximately 60%. On brown rice protein aggregates, it was observed that both disaccharides caused a decrease in adsorption capacity to 58.5%. Both disaccharides caused an increase in the adsorption capacity of proanthocyanidins on pea protein aggregates, approximately 76%. The same adsorption capacity was observed for rice protein aggregates; however, this value was lower than in the case without disaccharides. Regarding monomeric anthocyanins, the addition of disaccharide caused a decrease in adsorption capacities for both proteins in comparison to control samples (82 to 85%). Other types of complexations, i.e., firstly the complexation of disaccharides and proteins and then the addition of chokeberry juice, also caused changes in adsorption capacities. In comparison to the control sample, the adsorption capacity of total polyphenols increased with the addition of disaccharides on pea protein aggregates (approximately 66.4%), while a decrease occurred in the case of rice proteins (62%). Adsorption capacity of proanthocyanidins onto pea proteins highly increased when disaccharides were added, up to 84.4% and 86.3% in the case of trehalose and sucrose, respectively. In the case of rice proteins, disaccharides had a negative impact on adsorption capacities, thus adsorption of 72.4% and 77% was achieved for trehalose and sucrose addition, respectively. Adsorption capacities of monomeric anthocyanins onto pea proteins were 90.3% and 87.7% for trehalose and sucrose addition, respectively, i.e., sucrose had slightly lower adsorption capacity than the control sample, while trehalose had no effect. In the case of rice proteins, the opposite effect was observed, i.e., adsorption capacity of 92% was achieved for sucrose addition and 82% for trehalose addition.

### 2.2. Polyphenol Contents on Protein Aggregates

Firstly, the results of the evaluation of polyphenols and the antioxidant potential of chokeberry juice are presented in Table 2. Chokeberry juice contained 41.32 g/L of total polyphenols and 10.89 g/L of proanthocyanidins. Three anthocyanins were determined, cyanidin-3-galactoside, cyanidin-3-glucoside, and cyanidin-3-arabinoside. Cyanidin-3-galactoside was the dominant one (1589.24 mg/L), followed by cyanidin-3-arabinoside (589.36 mg/L), while cyanidin-3-glucoside was determined in the lowest concentration (107.22 mg/L). The dominant phenolic acid was chlorogenic acid (7.31 g/L), while nechlorogenic acid and an additional derivate of chlorogenic acid were determined in lower concentrations (3.36 g/L and 0.82 g/L, respectively). Also, quercetin was determined in juice at a concentration of 350.83 mg/L. Antioxidant potentials were 141.25 μmol/100 L, 175.21 μmol/100 L, 20.31 μmol/100 L, and 1048.08 μmol/100 L for DPPH, ABTS, FRAP, and CUPRAC assays, respectively.

Table 3 represents total polyphenols (TPC) and proanthocyanidin contents (PAC) of formulated protein aggregates. It is evident that the type of protein, addition of disaccharides and their type, as well as the way of complexation had an impact on the evaluated parameters. The RP+C aggregate had a higher TPC than PP+C (28.03 and 21.25 mg/g). Aggregates, when disaccharides were firstly complexed with pea protein, had a higher TPC (~26.2 mg/g) than PP+C, while complexation of all ingredients at the same time did not have an impact on the increase in TPC. In the case of rice protein aggregates, both combinations of complexation negatively affected TPC. TPC ranged from 22.41 to 24.04 mg/g. Regarding proanthocyanidin contents, the RP+C aggregate also had a higher content than PP+C (8.57 and 5.56 mg/g). All aggregates with the addition of disaccharides had a higher content of proanthocyanidins than the control sample. Especially, significantly higher PAC had aggregates when first disaccharides were complexed with pea proteins, ~9.3 mg/g, while PAC was ~6.4 mg/g when all ingredients were complexed at the same time. The same trend that was observed for TPC was observed for PAC in rice protein aggregates, i.e., all aggregates with disaccharide addition had lower PAC than the control one.

Concentrations of individual phenolic compounds on protein aggregates are presented in Table 4. From the results, it can be concluded that protein type, addition of disaccharides and their type had an impact on the concentration of individual phenolics. In addition, the order of individual ingredients during complexation influenced this parameter. Three anthocyanins were determined, cyanidin-3-galactoside, cyanidin-3-glucoside and cyanidin-3-arabinoside. Comparing the impact of protein type, it can be concluded that PP+C aggregate had a higher concentration of cyanidin-3-galactoside and cyanidin-3-glucoside than RP+C aggregate, while both aggregates contained the same concentration of cyanidin-3-arabinoside. PP+C aggregate (control sample) contained 1356.44 mg/kg of cyanidin-3-galactoside. The same concentration of this anthocyanin was determined on PP/T+C aggregate, while the other three aggregates had a lower concentration. When all ingredients were complexed at the same time, the aggregate with trehalose addition had a higher concentration of cyanidin-3-galactoside than one with sucrose addition (1184.74 and 1042.76 mg/kg, respectively). A different tendency was observed for brown rice aggregates. RP+C aggregate contained 1309.24 mg/kg of cyanidin-3-galactoside. Complexing all ingredients at the same time resulted in a higher concentration of this anthocyanin on RP+T+C aggregate (1402.63 mg/kg) and lower on RP+S+C aggregates (1226.36 mg/kg). The highest concentration of this anthocyanin was obtained when brown rice protein was first complexed with sucrose (1466.16 mg/kg). Contrary, when trehalose was first complexed with brown rice protein, the lowest binding of this anthocyanin was achieved (1054.83 mg/kg). Regarding cyanidin-3-glucoside, anthocyanin detected in the lower concentrations than the other two anthocyanins, it was determined that all combinations had a positive impact on the binding of this compound in contrast to control samples, i.e., PP+C and RP+C aggregates (32.72 and 18.22 mg/kg, respectively). On pea aggregates, the highest concentrations of this anthocyanin were obtained when disaccharides were first complexed with protein, with trehalose having a higher positive impact (69.76 mg/kg) than sucrose (57.41 mg/kg). When all ingredients were complexed together, again trehalose had a higher positive effect (47.78 mg/kg) on binding cyanidin-3-glucoside than sucrose (37.59 mg/kg). Contrary there was no difference between disaccharides impact on brown rice proteins when all ingredients were complexed at the same time (approximately 35 mg/kg). Cyanidin-3-arabinoside was determined in similar concentrations on both aggregates, PP+C and RP+C (approximately 460 mg/kg). Slightly higher concentration of this anthocyanin was determined when trehalose was first complexed with protein (472.75 mg/kg), and lower when sucrose was used (433.62 mg/kg). When all ingredients were complexed together, a lower concentration of cyanidin-3-arabinoside was evaluated in contrast to the PP+C aggregate. However, trehalose had a higher positive effect (402.88 mg/kg) on binding cyanidin-3-glucoside than sucrose (355.18 mg/kg). Interestingly, when the main carrier was rice protein, a different trend was achieved. Higher concentration of this anthocyanin was determined when sucrose was first complexed with protein (526.00 mg/kg), and lower when sucrose was used (345.43 mg/kg). When all ingredients were complexed together, the same trend was observed as for the PP+C aggregate, i.e., a lower concentration of cyanidin-3-arabinoside was evaluated in contrast to RP+C aggregates, and trehalose caused higher adsorption of this anthocyanin than sucrose (526.90 and 505.90 mg/kg, respectively).

On formulated aggregates, quercetin was detected. However, the positive impact of the addition of disaccharides during complexation was not detected. Comparing aggregates based on protein, it can be concluded that pea protein had a higher affinity for this compound than rice protein (304.02 and 271.23 mg/kg, respectively). All aggregates based on protein had a lower concentration of quercetin, and there was no difference between disaccharides’ impact, just between ways of complexation. When all ingredients were complexed together, a higher concentration (~297 mg/kg) of quercetin was observed than in cases when disaccharides were first complexed with the protein matrix (~284 mg/kg). In the case of rice protein, when all ingredients were complexed together, a higher concentration (~283 mg/kg) of quercetin was observed in contrast to RP+C.

Chlorogenic acid, neochlorogenic acid, and one more derivate of chlorogenic acid were detected in aggregates from a group of phenolic acids. Comparing protein matrices, there was no difference between them regarding the concentration of chlorogenic acid (~3.3 g/kg). All aggregates prepared based on pea protein had a lower concentration of chlorogenic acid than the control one. However, those prepared by firstly complexing disaccharides with protein contained a higher concentration (~2.85 g/kg) of this phenolic acid. The impact of the type of disaccharides was not detected. In the case of rice protein, a different trend was observed. Aggregates when protein, trehalose, and juice were complexed together, and when sucrose was first complexed with protein, had a higher concentration (~3.6 g/kg) of this acid than the control one, while aggregates in which trehalose was first complexed with protein had the lowest (~2.55 g/kg). The RP+C aggregate contained a higher concentration of neochlorogenic acid than PP+C (3.17 and 2.87 g/kg, respectively). All pea protein aggregates had a lower concentration of neochlorogenic acid than PP+C; however, higher concentrations were evaluated when disaccharides were first complexed with protein (2.24 and 2.12 g/kg) in contrast to aggregates when all ingredients were complexed at the same time (1.92 and 1.82 g/kg). In both cases, aggregates with trehalose contained a higher concentration of neochlorogenic acid. In the case of rice proteins, aggregates when protein, sucrose, and juice were complexed together and when trehalose was first complexed with protein, had a higher concentration (3.22 and 3.55 g/kg, respectively) of this acid than the control one. Regarding the derivate of chlorogenic acid, the PP+C aggregate had a much higher concentration than the RP+C aggregate (1.05 and 0.41 g/kg, respectively). The addition of disaccharides and the way of complexation did not affect the concentration of this phenolic acid. In the case of rice protein, when all ingredients were complexed at the same time and when sucrose was complexed first with protein, a higher concentration was evaluated in contrast to the control sample.

### 2.3. Antioxidant Potential of Formulated Protein Aggregates

Four frequently used assays (DPPH, FRAP, ABTS, and CUPRAC) were used for the evaluation of the antioxidant potential of formulated aggregates, and the results are presented in Table 5. Comparison of protein-based aggregates revealed that RP+C had higher antioxidant potential, determined with all assays than the PP+C protein. A similar behaviour was determined for all aggregates with the addition of disaccharides, regardless of the applied assay. When disaccharides were first complexed with pea protein, aggregates had higher values of antioxidant activity in contrast to aggregates when all ingredients were complexed at the same time. For rice protein aggregates in combination with disaccharides, all aggregates had lower antioxidant activity in contrast to the control sample. These results are in relation to total polyphenols, and especially proanthocyanidins content. Pea protein aggregates with disaccharides had higher total polyphenols content, especially proanthocyanidins than the control sample. While for rice protein aggregates with disaccharides, it was determined that those samples had lower total polyphenol and proanthocyanidin contents.

**Figure 2:**
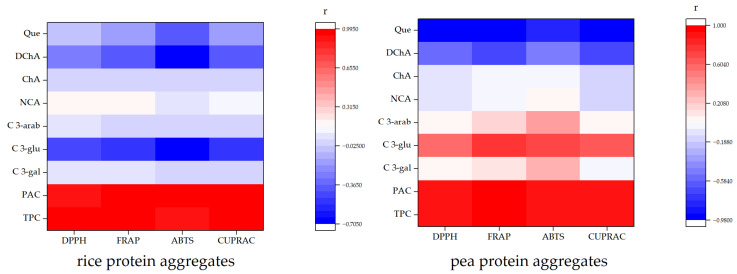
Heatmaps of correlation factors (r) between total polyphenols (TPC), proantocyanidins (PAC), and individual polyphenols (C 3-gal: cyanidin-3-galactoside; C 3-glu: cyanidin-3-glucoside; C 3-arab: cyanidin-3-arabinoside; NCA: neochlorogenic acid; ChA: chlorogenic acid; DChA: chlorogenic acid derivate; Que: quercetin) and antioxidant potential determined by DPPH, ABTS, FRAP, and CUPRAC assays.

The general rule is that the positive or negative linear correlation is stronger as the r is closer to +1 or −1, respectively. From the results (Figure 2), it can be seen that a strong positive correlation was obtained between total polyphenol contents and antioxidant activities determined by all assays (from 0.8915 to 0.9963), as well as between proanthocyanidin contents and antioxidant activities determined by all assays (from 0.8915 to 0.9963) for both protein aggregates. Regarding individual polyphenols, there was no strong positive correlation between any of the polyphenols and antioxidant potentials for both types of protein aggregates. For rice protein aggregates, there was no correlation between cyanidin-3-galactoside (from −0.012 to 0.0758) and cyanidin-3-arabinoside (from −0.0104 to 0.0516) with antioxidant potentials. Cyanidin-3-glucoside had a moderate negative correlation with antioxidant activities (from −0.4321 to −0.7037). No correlation of antioxidant potentials was also obtained with chlorogenic (from −0.02 to −0.0297) and neochlorogenic (from −0.0516 to −0.1738) acids. Derivate of chlorogenic acid showed a weak negative correlation (from −0.2767 to −0.414), except for ABTS antioxidant potential, which had a moderate negative correlation (−0.6526). Results for quercetin indicated that there is no correlation (from −0.0347 to −0.157), except for ABTS antioxidant potential, which had a weak negative correlation (−0.3918). Correlation factors were different for pea protein aggregates. No correlation was determined between cyanidin-3-galactoside (from 0.0025 to 0.1128) and cyanidin-3-arabinoside (from 0.0126 to 0.1526) with DPPH, FRAP, and CUPRAC antioxidant potentials, while for ABTS values, a weak correlation was achieved (0.3287 and 0.3832, respectively). For rice protein aggregates, a negative moderate correlation was observed, and for pea protein aggregates, a positive moderate correlation was obtained for cyanidin-3-glucoside (from 0.5501 to −0.7904). For chlorogenic (from −0.1778 to 0.0017) and neochlorogenic (from −0.1337 to 0.0128) acids, no correlation was observed, while for the derivate of chlorogenic acid, a negative moderate correlation (from −0.5103 to −0.7042) with antioxidant potentials was determined. Interestingly, for quercetin, a strong negative correlation was achieved for all antioxidant potential values (from −0.8103 to −0.9744).

**Figure 3:**
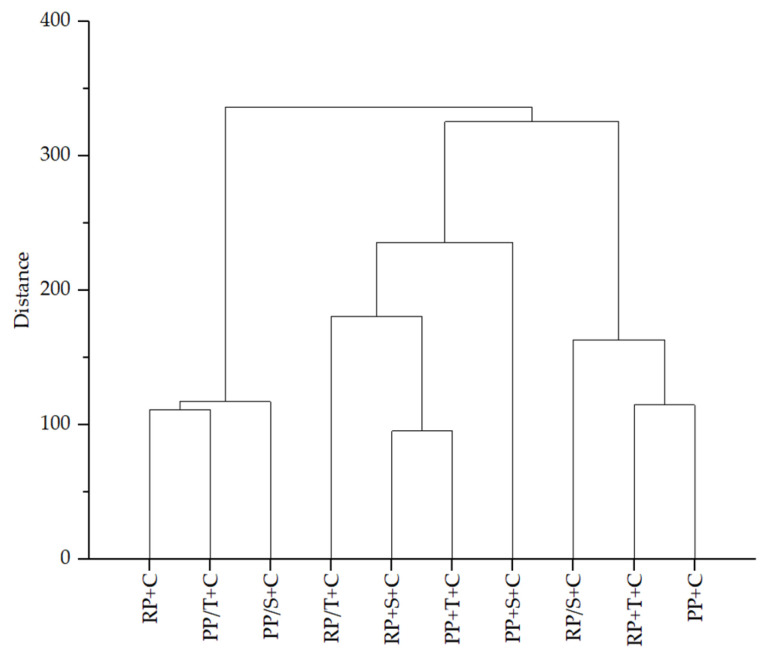
Dendrogram of similarity between all aggregates based on the results of polyphenols and antioxidant potentials (RP+C: complexation of rice proteins and chokeberry juice; RP/T+C: first complexation of rice proteins with trehalose and then with chokeberry juice; RP/S+C: first complexation of rice proteins with sucrose and then with chokeberry juice; RP+T+C: complexation of all ingredients (rice proteins, trehalose, and chokeberry juice) at the same time; RP+S+C: complexation of all ingredients (rice proteins, sucrose, and chokeberry juice) at the same time; PP+C: complexation of pea proteins and chokeberry juice; PP/T+C: first complexation of pea proteins with trehalose and then with chokeberry juice; PP/S+C: first complexation of pea proteins with sucrose and then with chokeberry juice; PP+T+C: complexation of all ingredients (pea proteins, trehalose, and chokeberry juice) at the same time; PP+S+C: complexation of all ingredients (pea proteins, sucrose, and chokeberry juice) at the same time.

A dendrogram was formulated based on the results of polyphenols and antioxidant potentials and is presented at Figure 3. According to the similarity, three main clusters were formed. The first contained RP+C and PP/T+C, the second RP+S+C and PP+T+C, and the third RP+T+C and PP+C aggregates. Very similar to the first cluster was the PP/S+C aggregate, while a lower similarity to the second and third clusters was observed for the RP/T+C and RP/S+C aggregates, respectively.

### 2.4. Recording of IR Spectra of Formulated Protein Aggregates

**Figure 4:**
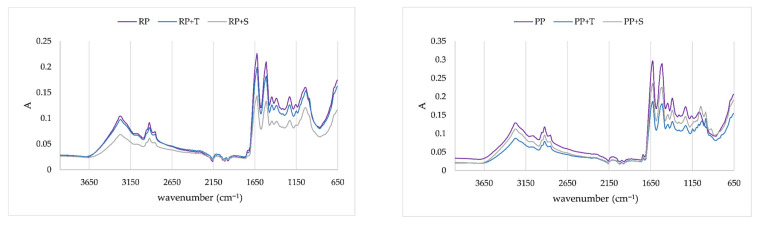
IR spectra of proteins and protein/disaccharide aggregates (RP: rice protein, PP: pea protein, RP+T: complexation of rice proteins trehalose; RP+S: complexation of rice proteins and sucrose; PP+T: complexation of pea proteins and trehalose; PP+S: complexation of pea proteins and sucrose).

**Figure 5:**
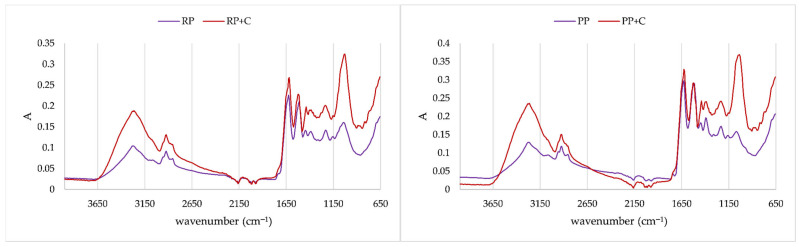
IR spectra of proteins and protein/polyphenol aggregates (RP: rice protein, PP: pea protein, RP+C: complexation of rice proteins and chokeberry juice; PP+C: complexation of pea proteins and chokeberry juice).

**Figure 6:**
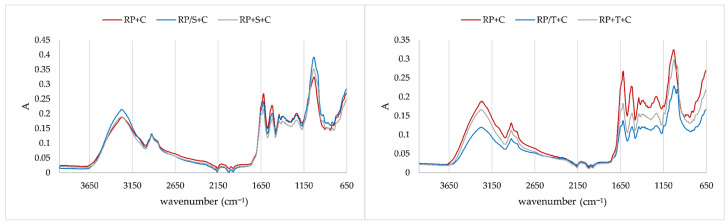
IR spectra of rice protein aggregates (RP+C: complexation of rice proteins and chokeberry juice; RP/T+C: first complexation of rice proteins with trehalose and then with chokeberry juice; RP/S+C: first complexation of rice proteins with sucrose and then with chokeberry juice; RP+T+C: complexation of all ingredients (rice proteins, trehalose, and chokeberry juice) at the same time; RP+S+C: complexation of all ingredients (rice proteins, sucrose, and chokeberry juice) at the same time).

**Figure 7:**
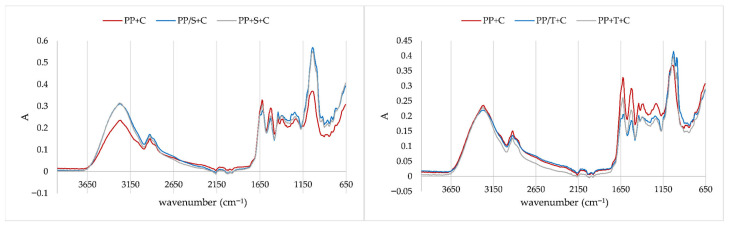
IR spectra of pea protein aggregates (PP+C: complexation of pea proteins and chokeberry juice; PP/T+C: first complexation of pea proteins with trehalose and then with chokeberry juice; PP/S+C: first complexation of pea proteins with sucrose and then with chokeberry juice; PP+T+C: complexation of all ingredients (pea proteins, trehalose, and chokeberry juice) at the same time; PP+S+C: complexation of all ingredients (pea proteins, sucrose, and chokeberry juice) at the same time).

IR spectra were recorded in order to prove interactions of polyphenols and proteins, as well as between polyphenols, disaccharides, and proteins, i.e., as proof of the existence of protein/polyphenols aggregates and protein/disaccharide/polyphenols aggregates. IR spectra of proteins and created aggregates are presented at Figure 4, Figure 5, Figure 6 and Figure 7. In order to obtain insight into whether proteins will interact with disaccharides, they were complexed together and prepared as protein aggregates with chokeberry juice. IR spectra of the obtained dry powders were recorded, and IR spectra are presented in Figure 4. On both types of proteins, disaccharides caused changes in the carbohydrate fingerprint region, which is from 1200 cm^−1^ to 950 cm^−1^ [33]. According to IR spectra, changes are more pronounced at pea protein aggregates, indicating that it is possible that a higher amount of disaccharides was adsorbed onto protein. On rice proteins, shoulder bands were created at 1103 cm^−1^ and 991 cm^−1^, both related to C–O and C–C bonds of sugar rings [34]. While IR spectra of rice proteins with trehalose and sucrose were similar, on pea proteins, differences between those two disaccharides were quite visible. Both disaccharides caused a shift in band 1160 cm^−1^ (related to CO stretching) on pea protein to 1148 cm^−1^ when trehalose was added and to 1125 cm^−1^ when sucrose was added. An additional band was formed, caused by both disaccharides at 1096 cm^−1^ related to C–O–C of carbohydrates [34]. Also, disaccharides caused the formation of bands at 1043 cm^−1^ for sucrose and 1028 cm^−1^ for trehalose, both related to C–O bending and C–OH bond of sugar ring [34].

In Figure 5, IR spectra of both proteins and corresponding aggregates prepared with chokeberry juice are presented to obtain insight into the impact of polyphenols on protein structure. Proteins are characterized by distinctive amide bands, which are situated in regions of 1700–1600 cm^−1^, 1600–1500 cm^−1^, 1380–1200 cm^−1^, and 3500–3300 cm^−1^. All of these regions are related to specific bonds, i.e., with amide I (C–O stretching), amide II (N–H bending and C–H stretching), amide III (N–H in plane bending coupled with C–N stretching), and amide A structures of proteins, respectively [35,36,37,38]. Secondary structures of proteins include α-helix (1658–1650 cm^−1^), β-sheet (1640–1610 cm^−1^), β-turn (1700–1660 cm^−1^), and random coil structure (1650–1640 cm^−1^) [37]. Adsorption of polyphenols on rice proteins caused changes in Amid I structure through a shift in the band at 1617 cm^−1^ on protein to 1625 cm^−1^ on aggregates, indicating changes in β-sheet. Both proteins exhibited changes in Amid II structure through a shift of band 1520 cm^−1^ on protein to 1513 cm^−1^ on aggregates. On both proteins, the band at 1438 cm^−1^ was quite sharp and changed into a less sharp band, indicating changes in CH_2_ stretching. Additionally, changes in band at 1380 cm^−1^ were observed due to changes in stretching of C–O, deformation of C–H, and deformation of N–H bonds. The slight shoulder at both proteins at 1274 cm^−1^ disappeared after the adsorption of polyphenols. Both changes indicate changes in Amid III structure. A slight change in band at 1150 cm^−1^ was observed, which indicated changes in stretching vibrations of hydrogen-bonding C–OH groups. On both proteins, the band at 3056 cm^−1^ related to the Amid B structure (N–H stretching) disappeared after adsorption of chokeberry polyphenols. The region from 3000 cm^−1^ to 2800 cm^−1^ represents hydrophobic interactions between proteins and polyphenols [39], and for both proteins, alternation in this region was observed by chokeberry polyphenols. On rice protein, bands at 2952 cm^−1^ and 2847 cm^−1^ were less pronounced after the adsorption of polyphenols, both bands related to C–H stretching and CH_3_ asymmetric stretching [34]. The band at 1750 cm^−1^ after adsorption of polyphenols was less pronounced, indicating changes in C–O stretching. Significant increase in band at 1030 cm^−1^ on both proteins occurred after adsorption of polyphenols, indicating changes of –CH_2_OH groups and the C–O stretching vibration coupled with C–O bending of the C–OH groups. Additional changes on both proteins occurred in the region from 900 cm^−1^ to 700 cm^−1^, indicating out-of-plane bending vibrations [34].

Comparison of IR spectra of rice protein/polyphenol aggregate with aggregates with the addition of disaccharides (Figure 6) revealed additional changes due to the presence of disaccharides in aggregates. The band at 1150 cm^−1^ (related to changes in stretching vibrations of hydrogen-bonding C–OH groups) in aggregates with sucrose addition disappeared, while it remained stable in trehalose aggregates. Sucrose aggregates also exhibited a slight shoulder and caused the formation of bands at 991 cm^−1^, which was also recorded on rice protein/sucrose aggregates. An additional band that was not detected on rice protein/sucrose aggregates was formed at 924 cm^−1^. Trehalose caused different changes in protein aggregates. A slight shoulder at 1074 cm^−1^ was created, and a band at 984 cm^−1^ was formed due to the presence of trehalose, changes related to carbohydrates fingerprint region. Both disaccharides caused changes in out-of-plane bending vibrations.

Comparison of IR spectra of pea protein/polyphenols aggregate with aggregates with disaccharides addition (Figure 7) also revealed additional changes due to the presence of disaccharides in these aggregates, as was the case with rice proteins. Sucrose protein aggregates also exhibited a slight shoulder and caused the formation of bands at 991 cm^−1^ and 924 cm^−1^, which were also recorded in rice protein aggregates. An additional band was formed at 1230 cm^−1^. Trehalose caused different changes in protein aggregates. A slight shoulder at 1058 cm^−1^ was created, along with a band at 991 cm^−1^. Both disaccharides caused changes in out-of-plane bending vibrations.

## 3. Discussion

The aim of this research was to establish the impact of the addition of disaccharides during the complexation of plant proteins with chokeberry juice on the encapsulation of polyphenols on selected protein carriers. Additionally, the effect of the selected methods of complexation on the encapsulation of polyphenols was investigated. Two methods were used: one included the complexation of all ingredients at the same time, while the other one was based first on the complexation of the disaccharide with protein matrix, and then this mixture was complexed with chokeberry juice. It was established that, next to the protein type and disaccharide type, the applied method of complexation had an impact on the encapsulation of chokeberry polyphenols.

Generally, the encapsulation of polyphenols onto proteins depends on the chemical characteristics of proteins and polyphenols [40]. Results of this research showed that the difference in the encapsulation of polyphenols on different types of proteins (rice protein and pea protein matrices) is quite pronounced. As was already mentioned, protein structure is very important in governing the binding of polyphenols. Selected protein matrices as carriers differ in their structure. Rice proteins contain mostly glutelin, next to prolamin, albumin, and globulin. An important characteristic of the glutelin fraction is its formation of a rigid matrix of lower solubility through the formation of disulfide bridges, which additionally creates surface locations for interactions with polyphenols [41]. The presence of side chain functional groups –NH_2_ and –COOH enables electrostatic interactions and the formation of hydrogen bonds with polyphenols [42], while aromatic amino acids enable π-π interactions [43]. Pea proteins are predominantly constructed of globulin, especially legumin and vicilin, which have pronounced hydrophobic regions. Side chain functional groups –NH_2_ and –COOH enable hydrophobic interactions, π-π interactions, and the formation of hydrogen bonds with polyphenols [25,44]. Aromatic amino acids additionally contribute to π-π interactions [45].

Our results showed that different polyphenols differ in their affinity towards proteins, and an additional impact of disaccharides was also observed. Structure of polyphenols, primarily the number and location of hydroxyl groups, affected the reactivity of flavonoids and governed their binding onto proteins [46]. Comparing the reactivity and intensity of binding of the selected polyphenols on soy protein matrix, Rawel et al. [46] observed that gallic acid had the highest affinity, followed by chlorogenic acid and quercetin. Lower affinity was observed for myricetin, caffeic acid, and kaempferol, while apigenin and flavone possessed considerably lower affinity for binding onto soy protein matrix. Sęczyk et al. [40] also investigated interactions between polyphenols and white bean proteins. They observed that chlorogenic and gallic acids had the highest affinity towards binding onto albumin. Lower affinity toward albumin was achieved by catechin and quercetin, while the lowest was observed for apigenin and ferulic acid. The results for globulin fraction slightly differ from those for albumin. Chlorogenic acid possessed the highest affinity, followed by catechin, gallic acid, and quercetin. Apigenin and ferulic acid had the lowest affinity towards globulin, as was the case with albumin [40]. The same authors also emphasized the importance of polyphenols composition in the matrix throughout the investigation of the binding of polyphenols from green coffee and green tea extracts on the mentioned protein fractions. Comparing the affinity of polyphenols from the extract towards the same protein fraction with the affinity of pure compounds, chlorogenic acid and catechin had a higher affinity than pure polyphenols. The fact that extracts included other polyphenols next to the mentioned ones highly affected their affinity towards proteins [40]. This could be a result of competition for the same binding locations on protein structure. The same effect can be observed in our study since chokeberry juice is a matrix containing different polyphenols, so competition between compounds for binding locations onto both selected proteins can be expected.

During complexation in our study, disaccharides were used, so their effect should be considered. Disaccharides in a complexation mixture can present additional binding sites for polyphenols, or interactions between disaccharides and proteins can result in the formation of new binding sites or loss of binding sites. It was already highlighted that protein matrices can contain other organic molecules like polysaccharides and can influence encapsulation efficiency [15,16,21,22,23,47,48,49]. Quercetin had a higher affinity for adsorption onto brown rice proteins (85% of proteins) than on almond (50% of proteins) protein matrix [22]. Comparing the adsorption affinity of glucosyl hesperidin onto proteins, the following order was achieved: pea proteins (85% of protein) > almond proteins (50% of proteins) > brown rice proteins (85% of protein) > pumpkin proteins (50% of proteins) [21]. Research on the encapsulation of cinnamic acid onto proteins revealed that the amount of applied protein matrix also significantly influenced its encapsulation. When 1% of the protein matrix was used for cinnamic acid encapsulation, the following order was achieved: pumpkin proteins (50% of proteins) > pea proteins (85% of protein) > almond proteins (50% of proteins); with an increase in the protein matrix, a different order was observed, i.e., pea proteins (85% of protein) > pumpkin proteins (50% of proteins) > almond proteins (50% of proteins) [23]. A study on the encapsulation of cranberry polyphenols onto proteins revealed that these polyphenols had the highest affinity towards defatted soy flour (50% of proteins), medium roasted peanut flour (50% of proteins), and hemp protein isolate (>70% of proteins). Additionally, the authors investigated the encapsulation of proanthocyanidins, and a different effect was observed; these compounds had the highest affinity towards hemp protein isolate (>70% of proteins) and the lowest to defatted soy flour (50% of proteins) [16]. In the study of Roopchand et al. [15], the adsorption of blueberry juice anthocyanins was the highest on defatted soy flour (47% of proteins), followed by white whole-wheat flour (13% of proteins) and brown rice flour (8.6% of proteins), and the lowest on corn flour (5.3% of proteins). All of these studies proved that the type of proteins, their content, and additional organic compounds are responsible for encapsulation of different types of polyphenols. Based on our results, it was concluded that disaccharides, as smaller carbohydrate molecules, were also involved in the binding of chokeberry polyphenols onto rice and pea protein matrices.

In addition to the interactions between proteins and phenolics, and between proteins, disaccharides, and phenolics, an additional stacking effect can be anticipated, i.e., interactions between anthocyanins can occur with anthocyanins already attached on proteins and disaccharides, as it was stated in other studies [26,50]. The presence of larger biopolymers, such as proteins and smaller molecules, such as disaccharides, in complexation mixtures provides tighter molecular packaging. Larger proteins can create gaps that are usually filled with smaller molecules like disaccharides [51]. This can have a dual effect; on the one side, it can help in capturing certain polyphenols, while on the other side, it can retard the number of binding sites for the other polyphenols, and this effect was noticeable in this study.

The interaction between utilized ingredients during complexation can be one of the mechanisms for capturing polyphenols. An additional mechanism involved in the capture of polyphenols could be the alternation of water dynamics by disaccharides, since there was a substantial amount of water in the complexation mixture. Sucrose and trehalose are chemical isomers; however, they differ in certain characteristics regarding their behaviour in water. On the basis of the possibility to form clusters, the hydration number, the gyration radius, and the glycosidic dihedral angles, it was concluded that trehalose creates more homogenous water solutions in contrast to sucrose [52]. Trehalose has a larger impact on water structure, which is also seen through its binding with more water molecules than sucrose, resulting in a more pronounced destructuring impact on water [53] and consequently changing water’s impact on polyphenols.

Accessibility of water in the complexation mixture had an impact on its interactions with the protein mixture, disaccharides, and phenolic compounds, as well as on impact on their interactions between themselves. The presence of water in the system provides conditions that represent the initiating power for carrying out diffusion-dependent reactions. Water in higher amounts causes deterioration of sensitive compounds, like phenolics, since molecular mobility is increased and oxidation reactions are enhanced. Within phenolic compounds, anthocyanins are particularly sensitive to water. A consequence of the hydrolysis of the glycosidic bond in the structure of anthocyanins is the formation of anthocyanidins, which are known for even higher instability than anthocyanins. This reaction is usually followed by the opening of the pyrilium ring that results in the creation of chalcones and brown final components [54]. Next to these reactions, the presence of oxygen results in a rise in the oxidation rate of anthocyanins. The previously ascribed impact of disaccharides on the behaviour in water can additionally cause changes in the impact of water.

Different values of antioxidant potential of protein aggregates were achieved, which depend on the applied assays as well as encapsulated compounds on carriers. Assays used for the estimation of antioxidant potential possess a specific reaction mechanism. The reaction mechanism of the DPPH assay includes the transfer of a hydrogen atom and the transfer of a proton-coupled electron in reactions with polyhydroxy aromatic compounds [55]. ABTS reaction mechanism includes the transfer of a hydrogen atom, the transfer of a single electron, or the SPLET (sequential proton loss electron transfer) mechanism. The former mechanism is particularly pronounced in water or alcohol; thus, in those media, phenols are ionized to phenoxide anions, which act as electron donors. ABTS free radicals are characteristic by their ability to react with lipophilic and hydrophilic compounds [55]. The reaction mechanism of the other two assays is different. FRAP assay involves the reduction reaction of the complex of ferric ion (Fe^3+^)-ligand to ferrous (Fe^2+^) complex [56]. The mechanism of action of the CUPRAC assay involves the reduction reaction of cupric (Cu^2+^) to cuprous ion (Cu^+^) [57]. Since antioxidant potential is one of the crucial properties of polyphenols that is mainly determined by their chemical structure, different values for different assays were achieved.

The overall antioxidant potential of the matrix that represents the mixture of various polyphenols can be additive, synergistic, or antagonistic. This phenomenon mainly depends on diversification in the structure of polyphenols, defining that the number and location of OH-groups and OCH_3_-groups on the phenolic rings are the main factors in charge of their contribution to antioxidant potential. Next to these factors, for the overall manifestation of the antioxidant potential of the complex matrix, additional factors have to be taken into account. Some of those factors are the concentration of phenolics, the ratio among them, their tendency for dissociation and ionization, their ability to undergo intramolecular and/or intermolecular interactions, as well as matrix interference [55,58,59]. This phenomenon is especially visible from the results of the correlation calculation between individual phenolic compounds and values of antioxidant potential. A strong correlation was only achieved between proanthocyanidins and antioxidant potential, without a strong correlation between other phenolics and antioxidant potential. Antioxidant potential of proanthocyanidins is ascribed to the existence of a large number of –OH groups, but also to their particular stereochemical structural features, the synergistic impact between the polymers, and polymerization degree. It has been determined that proanthocyanidins with a low polymerization degree possess stronger antioxidant potential due to a larger number of available –OH groups. With the increase in polymerization degree, changes in structure occur, i.e., molecular interspaces of proanthocyanidins become tighter, obstructing the availability and activity of –OH groups [60]. Chokeberries are a rich source of B-type proanthocyanidins [61].

Recording of IR spectra showed that there were interactions between proteins and chokeberry polyphenols, as well as proteins, disaccharides, and polyphenols. The binding of polyphenols on proteins through the recording of IR spectra was determined in many other studies. The interactions between those compounds can be of a covalent and/or non-covalent nature [49,62,63]. Non-covalent ones include interactions through hydrogen bonds, hydrophobic association, van der Waals forces, and electrostatic attraction. However, the most significant non-covalent interactions between polyphenols and proteins are hydrophobic interactions and hydrogen bonds [64]. The formation of soybean protein–anthocyanin conjugates [65] and soy protein isolate–anthocyanin conjugates [66] was proved by IR spectra recording. Covalent interactions of β-lactoglobulin with caffeoylquinic acids [67] caused changes in the amid region, i.e., reduction in α-helix and β-sheet and an increase in random coil and β-turn. Structural changes in brown rice proteins after adsorption of raspberry juice polyphenols occurred [68]. Adsorption of quercetin on brown rice proteins and almond proteins also caused changes in amid regions and hydrophobic interactions [22], and it was determined for binding cinnamic acid onto pea, almond, and pumpkin proteins [23].

## 4. Materials and Methods

### 4.1. Ingredients for the Creation of Protein Aggregates

Chokeberry juice was prepared by the pressing of washed fruits, which were cultivated in an area at 46.176799° N 16.333695° E and harvested in 2023. In order to obtain a stable system for further utilization, the mass after pressing was filtered and thermally treated for 3 min at 90 °C for inactivation of enzymes and microorganisms. For the creation of protein aggregates, two protein matrices were used, and two disaccharides, sucrose (99.8%) and trehalose (99.5%), were utilized. Pea protein matrix was obtained from Blesterfeld (Hamburg, Germany), while rice protein matrix was obtained from Biovega (Zagreb, Croatia). Hayashibara Co. (Nagase group, Okayama, Japan) donated trehalose, while sucrose was purchased from Gram-mol (Zagreb, Croatia). For spectrophotometric analysis, several solvents and specific reagents were utilized. Hydrochloric acid (37%) and methanol were products of Carlo Erba Reagents (Sabadell, Spain), while acetic acid (>99.5%) of Alkaloid (Skopje, North Macedonia). From Gram-mol (Zagreb, Croatia), ammonium acetate, calcium chloride, potassium chloride, sodium acetate, and ethanol were purchased. From T.T.T. (Sveta Nedelja, Croatia), sodium carbonate was obtained, while Folin–Ciocalteu reagent and potassium persulfate were obtained from Kemika (Zagreb, Croatia). 2,4,6-tri(2-pyridyl)-s-triazine, neocuproine, and cupric chloride were products of Acros Organic (Geel, Belgium). 2,2-diphenyl-1-picrylhydrazil, 2,2′-azino-bis(3-ethylbenzothiazoline-6-sulfonic acid) diammonium salt, 4-dimethyl-amino-cinnamaldehyd, and trolox were obtained from Sigma-Aldrich (St. Louis, MO, USA). For HPLC analysis, two mobile phases were purchased, both HPLC grade, orthophosphoric acid from Fisher Scientific (Loughborough, UK) and methanol from J.T. Baker (Deventer, The Netherlands). Standards of procyanidin B2, chlorogenic acid, and quercetin were obtained from Sigma-Aldrich (St. Louis, MO, USA), while cyanidin-3-galactoside, cyanidin-3-arabinoside, and neochlorogenic acid were obtained from Extrasynthese (Genay, France).

### 4.2. Creation of Protein Aggregates

For the preparation of aggregates, pea or rice protein matrices (containing approximately 85% and 80% of proteins, respectively), disaccharides (sucrose and trehalose), and chokeberry juice were selected. For all aggregates, the amount of chokeberry juice was constant, while the ratio protein:disaccharide was 1:1 for protein/disaccharides/phenolics aggregates. Protein aggregates were prepared by complexation through stirring of 2 g of protein matrix with 10 mL of distilled water and 20 mL of chokeberry juice using a magnetic stirrer at room temperature for 15 min. For protein/disaccharides/phenolics aggregates, two methods of complexation were used. The first included complexation of all ingredients at the same time, and the second was based first on complexation of disaccharides with protein matrix and then with chokeberry juice. For the first complexation, 2 g of protein matrix, 2 g of disaccharide, 10 mL of distilled water, and 20 mL of chokeberry juice were stirred at a magnetic stirrer at room temperature for 15 min. RP+T+C, RP+S+C, PP+T+C, and PP+S+C were created. For the second complexation, 2 g of protein matrix, 2 g of disaccharide and 10 mL of distilled water were first stirred at a magnetic stirrer at room temperature for 15 min, afterwards 20 mL of chokeberry juice was added and stirred for another 15 min. RP/T+C, RP/S+C, PP/T+C, and PP/S+C were created. After stirring the ingredients, the mixtures were centrifuged for 15 min at 8000 rpm. The obtained supernatants were separated from the precipitates, which were further freeze-dried in order to obtain dry powder. Foremost, precipitates were frozen for 24 h at −18 °C. Freeze-drying was performed in a laboratory freeze-dryer (Christ Freeze Dryer, Alpha 1-4, Osterode am Harz, Germany). Selected terms for freeze-drying of precipitates were adjusted to −55 °C as the temperature of freezing; from −35 °C to 0 °C as the temperature of sublimation under a vacuum of 0.220 mbar; from 0 °C to 21 °C as the temperature of the isothermal desorption under a vacuum of 0.060 mbar. Samples were freeze-dried for 6 h, resulting in the creation of dried powder. Coding of formulated protein aggregates is presented in Table 1.

### 4.3. Extraction of Polyphenols from Protein Aggregates

For the extraction of polyphenols, approximately 0.2 g of the freeze-dried protein aggregates was weighed in a plastic cuvette and mixed with 5 mL of acidified methanol (ratio of methanol and HCl was 99:1). The obtained mixture was treated in an ultrasonic bath for 15 min. After that, the mixture was centrifuged for 15 min at 4000 rpm. After that, the supernatant was collected, while the precipitate was extracted once more with 2 mL of acidified methanol. The second supernatant was combined with the first one, and the obtained extract was used for spectrophotometric analysis and the estimation of individual polyphenols.

### 4.4. Estimation of Total Polyphenols

Total polyphenols were determined using a Folin–Ciocalteu assay previously described in detail by Singleton and Rossi [69]. The experiments were conducted in triplicate, expressing results as mg of gallic acid equivalents per g of protein aggregate (mg GAE/g).

### 4.5. Estimation of Total Proanthocyanidins

Total proanthocyanidins were determined using an assay described in detail by Prior et al. [70]. The experiments were conducted in triplicate, expressing results as mg of procyanidin B2 equivalent per g of the protein aggregate (mg PB2E/g).

### 4.6. Estimation of Monomeric Anthocyanins

The monomeric anthocyanins were determined by the application of the pH differential method described by Giusti and Wrolstad [71].

### 4.7. Calculation of Adsorption Capacity

Calculation of adsorption capacity [72] was conducted for total polyphenols, proanthocyanidins, and monomeric anthocyanins according to the following equation:

AC (%) = ((CM − ST)/CM) × 100

where AC is the adsorption capacity, CM is the amount of compounds in the complexation mixture (total polyphenols, proanthocyanidins, or monomeric anthocyanins), and ST is the amount of compounds in supernatant (total polyphenols, proanthocyanidins, or monomeric anthocyanins).

### 4.8. Estimation of Individual Polyphenols by High-Performance Liquid Chromatography (HPLC)

Polyphenols were identified and quantified throughout the application of an Agilent HPLC system 1260 Infinity II (Agilent Technology, Santa Clara, CA, USA) equipped with Poroshell 120 EC C-18 column (4.6 × 100 mm, 2.7 μm), quaternary pump, diode array detector (DAD), and a vial sampler. The protocol was described in detail by Buljeta et al. [73]. The calibration curves were prepared for cyanidin-3-galactoside, cyanidin-3-arabinoside, quercetin, neochlorogenic, and chlorogenic acids at different concentrations. UV/Vis spectra were recorded in the range of 190 to 600 nm. Identification of the compounds was performed through comparison of retention times and the spectrum of peaks in extracts with those of the polyphenol standard. The experiments were conducted in duplicates.

### 4.9. Estimation of Antioxidant Potential

Antioxidant assays selected for estimation of antioxidant potential were DPPH, ABTS, FRAP, and CUPRAC. Absorbance of samples was recorded on a spectrophotometer (Cary 60, UV-Vis, Agilent Technologies, Santa Clara, CA, USA). The experiments were conducted in triplicate, expressing results as μmol of Trolox equivalents per 100 mL of sample (μmol TE/100 mL). Brand-Williams et al. [74] described the DPPH assay in detail. Arnao et al. [75] described the ABTS assay in detail. The ferric reducing ability of plasma (FRAP) assay was described in detail by Benzie and Strain [76]. Apak et al. [77] described the cupric reducing antioxidant capacity (CUPRAC) assay in detail.

### 4.10. Fourier Transform Infrared Spectroscopy–Attenuated Total Reflectance (FTIR-ATR) Analysis

Recording of infrared spectra of the samples was carried out on FTIR-ATR (Cary 630; Agilent, Santa Clara, CA, USA), which is equipped with MicroLab Expert (Agilent, Santa Clara, CA, USA) software. The recording of IR spectra ranged from 4000 to 600 cm^−1^ was selected.

### 4.11. Statistical Analysis of Obtained Results

All results are presented as mean value ± standard deviation (SD) of three replicates. Statistica 13.1 (StatSoft, Tulsa, OK, USA) software was used for one-way ANOVA and Fisher’s LSD test. OriginPro 2016 (OriginLab Corporation, Northampton, MA, USA) software was used for hierarchical cluster analysis and heatmaps evaluation of the obtained data.

## 5. Conclusions

Results of this research showed that disaccharides, as well as complexation methods, affected the encapsulation of polyphenols on proteins. Comparing protein matrix, higher amounts of total polyphenols and proanthocyanidins were determined on rice protein aggregates. While the addition of disaccharides, regardless of utilized complexation method, had a negative effect on the encapsulation of polyphenols on rice proteins, they had a positive effect on the encapsulation of polyphenols on pea proteins. Based on our results, for further application, rice protein aggregates and pea protein aggregates would be recommended. Created protein aggregates can be used as functional food additives in order to enrich products (such as bakery products, dairy products, fruit products) with plant proteins and chokeberry polyphenols, additionally promoting antioxidant stability of the product. These aggregates can be used for colour modification as well due to the high concentration of anthocyanins. However, additional research should be conducted involving the investigation of the behaviour of these aggregates in food products.

## Figures and Tables

**Table 1:** Explanation of the samples coding.

Sample	Method of Preparation
RP+C	Complexation of rice proteins and chokeberry juice
RP/T+C	First complexation of rice proteins with trehalose and then with chokeberry juice
RP/S+C	First complexation of rice proteins with sucrose and then with chokeberry juice
RP+T+C	Complexation of all ingredients (rice proteins, trehalose, and chokeberry juice) at the same time
RP+S+C	Complexation of all ingredients (rice proteins, sucrose, and chokeberry juice) at the same time
PP+C	Complexation of pea proteins and chokeberry juice
PP/T+C	First complexation of pea proteins with trehalose and then with chokeberry juice
PP/S+C	First complexation of pea proteins with sucrose and then with chokeberry juice
PP+T+C	Complexation of all ingredients (pea proteins, trehalose, and chokeberry juice) at the same time
PP+S+C	Complexation of all ingredients (pea proteins, sucrose, and chokeberry juice) at the same time

**Table 2:** Results of polyphenols and antioxidant potential of chokeberry juice.

TPC (g GAE/L)		PAC (g PB2E/L)		DPPH (μmol TE/100 L)			ABTS (μmol TE/100 L)		FRAP (μmol TE/100 L)		CUPRAC (μmol TE/100 L)	
41.32 ± 0.11		10.89 ± 0.04		141.25 ± 0.55			175.21 ± 0.45		20.31 ± 0.12		1048.08 ± 2.31	
**Individual polyphenols**												
C 3-gal (mg/L)		C 3-glu (mg/L)		C 3-arab (mg/L)	NCA (g/L)		ChA (g/L)		DChA (g/L)		Que (mg/L)	
1589.24 ± 10.87		107.22 ± 1.24		589.36 ± 7.43	3.36 ± 0.89		7.31 ± 0.30		0.82 ± 0.21		350.83 ± 4.21	

TPC: total polyphenols; PAC: proanthocyanidins; GAE: gallic acid equivalents; PB2E: procyanidin B2 equivalents; C 3-gal: cyanidin-3-galactoside; C 3-glu: cyanidin-3-glucoside; C 3-arab: cyanidin-3-arabinoside; NCA: neochlorogenic acid; ChA: chlorogenic acid; DChA: chlorogenic acid derivate; Que: quercetin.

**Table 3:** Total polyphenol (TPC) and proanthocyanidin contents (PAC) on formulated protein aggregates without and with the addition of disaccharides.

Sample	TPC (mg GAE/g)	PAC (mg PB2E/g)
RP+C	28.03 ± 0.32 ^a^	8.57 ± 0.03 ^b^
RP/T+C	23.56 ± 0.39 ^d^	6.85 ± 0.02 ^d^
RP/S+C	24.04 ± 0.04 ^c^	7.28 ± 0.00 ^c^
RP+T+C	22.41 ± 0.27 ^e^	6.36 ± 0.02 ^e^
RP+S+C	23.18 ± 0.39 ^d^	6.36 ± 0.00 ^e^
PP+C	21.25 ± 0.08 ^f^	5.56 ± 0.14 ^f^
PP/T+C	26.09 ± 0.06 ^b^	9.18 ± 0.09 ^a^
PP/S+C	26.44 ± 0.17 ^b^	9.45 ± 0.09 ^a^
PP+T+C	21.42 ± 0.16 ^f^	6.37 ± 0.04 ^e^
PP+S+C	22.19 ± 0.16 ^e^	6.40 ± 0.01 ^e^

RP+C: Complexation of rice proteins and chokeberry juice; RP/T+C: first complexation of rice proteins with trehalose and then with chokeberry juice; RP/S+C: first complexation of rice proteins with sucrose and then with chokeberry juice; RP+T+C: complexation of all ingredients (rice proteins, trehalose, and chokeberry juice) at the same time; RP+S+C: complexation of all ingredients (rice proteins, sucrose, and chokeberry juice) at the same time; PP+C: complexation of pea proteins and chokeberry juice; PP/T+C: first complexation of pea proteins with trehalose and then with chokeberry juice; PP/S+C: first complexation of pea proteins with sucrose and then with chokeberry juice; PP+T+C: complexation of all ingredients (pea proteins, trehalose, and chokeberry juice) at the same time; PP+S+C: complexation of all ingredients (pea proteins, sucrose, and chokeberry juice) at the same time; GAE: gallic acid equivalents; PB2E: procyanidin B2 equivalents. Data marked with different letters (a–f) in the same column are statistically different by ANOVA and Fisher’s (LSD) test, with *p* < 0.05.

**Table 4:** Concentration of individual polyphenols on formulated protein aggregates without and with the addition of disaccharides, determined using HPLC analysis.

Sample	C 3-gal (mg/kg)	C 3-glu (mg/kg)	C 3-arab (mg/kg)	NCA (g/kg)	ChA (g/kg)	DChA (g/kg)	Que (mg/kg)
RP+C	1309.24 ± 17.54 ^d^	18.22 ± 4.99 ^g^	455.36 ± 11.63 ^b^	3.17 ± 0.30 ^b^	3.20 ± 0.30 ^b^	0.407 ± 0.092 ^d^	271.23 ± 5.95 ^d^
RP/T+C	1054.83 ± 16.94 ^g^	23.32 ± 2.41 ^g^	345.43 ± 6.21 ^e^	2.55 ± 0.01 ^e^	2.56 ± 0.04 ^d^	0.430 ± 0.040 ^d^	261.34 ± 2.51 ^e^
RP/S+C	1466.16 ± 12.05 ^a^	34.25 ± 1.49 ^e^	526.00 ± 10.95 ^a^	3.55 ± 0.05 ^a^	3.72 ± 0.03 ^a^	0.764 ± 0.045 ^c^	269.25 ± 1.49 ^d^
RP+T+C	1402.63 ± 6.91 ^b^	35.98 ± 1.86 ^de^	505.90 ± 9.41 ^a^	3.22 ± 0.04 ^b^	3.60 ± 0.02 ^b^	0.878 ± 0.041 ^b^	285.58 ± 4.62 ^c^
RP+S+C	1226.36 ± 17.83 ^e^	34.41 ± 0.43 ^e^	431.88 ± 7.58 ^c^	2.63 ± 0.01 ^d^	3.07 ± 0.05	0.831 ± 0.043 ^bc^	281.78 ± 2.79 ^c^
PP+C	1356.44 ± 20.23 ^c^	32.72 ± 1.55 ^e^	465.87 ± 16.51 ^b^	2.87 ± 0.05 ^c^	3.49 ± 0.13 ^b^	1.05 ± 0.070 ^a^	304.02 ± 2.04 ^a^
PP/T+C	1356.59 ± 15.65 ^c^	69.76 ± 1.66 ^a^	472.73 ± 7.96 ^b^	2.24 ± 0.05 ^f^	2.90 ± 0.03 ^c^	0.941 ± 0.020 ^a^	284.34 ± 0.81 ^c^
PP/S+C	1253.95 ± 21.63 ^e^	57.41 ± 0.93 ^b^	433.62 ± 7.51 ^c^	2.12 ± 0.08 ^f^	2.82 ± 0.06 ^c^	0.976 ± 0.061 ^a^	284.06 ± 0.20 ^c^
PP+T+C	1184.74 ± 2.59 ^e^	47.78 ± 0.66 ^c^	402.88 ± 1.83 ^d^	1.20 ± 0.03 ^h^	2.68 ± 0.01 ^d^	0.993 ± 0.012 ^a^	299.33 ± 2.53 ^ab^
PP+S+C	1042.76 ± 6.07 ^g^	37.59 ± 0.54 ^d^	355.18 ± 1.57 ^e^	1.83 ± 0.04 ^g^	2.62 ± 0.02 ^d^	1.01 ± 0.008 ^a^	296.64 ± 0.54 ^b^

RP+C: Complexation of rice proteins and chokeberry juice; RP/T+C: first complexation of rice proteins with trehalose and then with chokeberry juice; RP/S+C: first complexation of rice proteins with sucrose and then with chokeberry juice; RP+T+C: complexation of all ingredients (rice proteins, trehalose, and chokeberry juice) at the same time; RP+S+C: complexation of all ingredients (rice proteins, sucrose, and chokeberry juice) at the same time; PP+C: complexation of pea proteins and chokeberry juice; PP/T+C: first complexation of pea proteins with trehalose and then with chokeberry juice; PP/S+C: first complexation of pea proteins with sucrose and then with chokeberry juice; PP+T+C: complexation of all ingredients (pea proteins, trehalose, and chokeberry juice) at the same time; PP+S+C: complexation of all ingredients (pea proteins, sucrose, and chokeberry juice) at the same time; C 3-gal: cyanidin-3-galactoside; C 3-glu: cyanidin-3-glucoside; C 3-arab: cyanidin-3-arabinoside; NCA: neochlorogenic acid; ChA: chlorogenic acid; DChA: chlorogenic acid derivate; Que: quercetin. Data marked with different letters (a–h) in the same column are statistically different by ANOVA and Fisher’s (LSD) test, with *p* < 0.05.

**Table 5:** Antioxidant potential (µmol TE/100 g) of formulated protein aggregates without and with the addition of disaccharides evaluated by DPPH, FRAP, ABTS, and CUPRAC assays.

Sample	DPPH	FRAP	ABTS	CUPRAC
RP+C	97.93 ± 1.03 ^a^	17.29 ± 0.08 ^a^	168.34 ± 3.74 ^a^	1449.88 ± 0.62 ^b^
RP/T+C	85.13 ± 0.43 ^e^	14.43 ± 0.00 ^d^	141.55 ± 0.75 ^d^	1193.81 ± 45.5 ^g^
RP/S+C	89.43 ± 0.60 ^c^	15.41 ± 0.06 ^c^	144.79 ± 0.70 ^c^	1289.29 ± 2.17 ^e^
RP+T+C	85.56 ± 0.44 ^e^	13.85 ± 0.3 ^e^	119.40 ± 1.95 ^f^	1126.68 ± 1.06 ^h^
RP+S+C	86.26 ± 1.42 ^e^	14.02 ± 0.2 ^de^	126.25 ± 1.18 ^e^	1189.01 ± 7.28 ^g^
PP+C	81.75 ± 2.17 ^f^	12.34 ± 0.15 ^g^	121.92 ± 1.32 ^f^	1221.92 ± 6.38 ^f^
PP/T+C	93.44 ± 0.89 ^b^	16.82 ± 0.00 ^b^	156.55 ± 2.31 ^b^	1532.64 ± 15.09 ^a^
PP/S+C	98.12 ± 0.66 ^a^	16.91 ± 0.53 ^ab^	158.29 ± 1.07 ^b^	1548.58 ± 17.55 ^a^
PP+T+C	82.70 ± 1.64 ^f^	12.79 ± 0.12 ^g^	114.45 ± 2.72 ^g^	1265.86 ± 7.49 ^d^
PP+S+C	87.48 ± 0.08 ^d^	13.22 ± 0.15 ^f^	112.43 ± 2.02 ^g^	1401.11 ± 6.86 ^c^

RP+C: Complexation of rice proteins and chokeberry juice; RP/T+C: first complexation of rice proteins with trehalose and then with chokeberry juice; RP/S+C: first complexation of rice proteins with sucrose and then with chokeberry juice; RP+T+C: complexation of all ingredients (rice proteins, trehalose, and chokeberry juice) at the same time; RP+S+C: complexation of all ingredients (rice proteins, sucrose, and chokeberry juice) at the same time; PP+C: complexation of pea proteins and chokeberry juice; PP/T+C: first complexation of pea proteins with trehalose and then with chokeberry juice; PP/S+C: first complexation of pea proteins with sucrose and then with chokeberry juice; PP+T+C: complexation of all ingredients (pea proteins, trehalose, and chokeberry juice) at the same time; PP+S+C: complexation of all ingredients (pea proteins, sucrose, and chokeberry juice) at the same time; TE: Trolox equivalents. Data marked with different letters (a–h) in the same column are statistically different by ANOVA and Fisher’s (LSD) test, with *p* < 0.05.

## Data Availability

The original contributions presented in this study are included in the article. Further inquiries can be directed to the corresponding author.
